# Novel scaffold based graphene oxide doped electrospun iota carrageenan/polyvinyl alcohol for wound healing and pathogen reduction: in-vitro and in-vivo study

**DOI:** 10.1038/s41598-021-00069-0

**Published:** 2021-10-14

**Authors:** Marwa H. Gouda, Safaa M. Ali, Sarah Samir Othman, Samia A. Abd Al-Aziz, Marwa M. Abu-Serie, Noha A. Elsokary, Noha A. Elessawy

**Affiliations:** 1 Polymer Materials Research Department, Advanced Technology and New Materials Research Institute, City of Scientific Research and Technological Applications (SRTA-City), New Borg El-Arab City, Alexandria, 21934 Egypt; 2 Nucleic Acid Research Department, Genetic Engineering and Biotechnology Research Institute, City of Scientific Research and Technological Applications (SRTA-City), Alexandria, Egypt; 3 Pharmaceutical Bioproducts Research Department, Genetic Engineering and Biotechnology Research Institute, City of Scientific Research and Technological Applications (SRTA-City), Alexandria, Egypt; 4 Department of Medical Biotechnology, Genetic Engineering, and Biotechnology Research Institute, City of Scientific Research and Technological Applications (SRTA-City), Alexandria, Egypt; 5 Pharmaceutical and Fermentation Industries Development Centre, City of Scientific Research and Technological Applications (SRTA-City), Alexandria, Egypt; 6 Advanced Technology and New Materials Research Institute, City of Scientific Research and Technological Applications (SRTA-City), Alexandria, Egypt

**Keywords:** Biochemistry, Biotechnology, Medical research, Materials science, Nanoscience and technology

## Abstract

Wound healing is a complicated multicellular process that involves several kinds of cells including macrophages, fibroblasts, endothelial cells, keratinocytes and platelets that are leading to their differentiation towards an anti-inflammatory response for producing several chemokines, cytokine and growth factors. In this study, electrospun nanofiber scaffold named (MNS) is composed of polyvinyl alcohol (PVA)/iota carrageenan (IC) and doped with partially reduced graphene oxide (prGO) that is successfully synthesized for wound healing and skin repair. The fabricated MNS was tested in case of infection and un-infection with *E. coli* and *Staphylococcus* and in both of the presence and in the absence of yeast as a natural nutritional supplement. Numerous biochemical parameters including total protein, albumin, urea and LDH, and hematological parameters were evaluated. Results revealed that the MNS was proved to be effective on most of the measured parameters and had exhibited efficient antibacterial inhibition activity. Whereas it can be used as an effective antimicrobial agent in wound healing, however, histopathological findings confirmed that the MNS caused re-epithelialization and the presence of yeast induced hair follicles growth and subsequently it may be used to hide formed head wound scar.

## Introduction

The human body's main organ is the skin. It acts as an essential part in various processes, such as hydration, initialization of vitamin D production, defense of pathogens, chemicals and thermal control. Its damage may be life-threatening. Nevertheless, skin wound contamination is a major clinical problem which results in both of immune responses and systemic inflammatory response syndrome (SIRS)^1^. In addition, other serious diseases such as infections of the blood stream which may be caused by *Staphylococcus* and *Escherichia coli*^2^ are clearly documented. On the other hand, antibiotics such as Gentamicin are widely to beused in wound treatment^3^. However, the over use of these antibiotics could result in severe side effects such as increasing the risk of antimicrobial resistance that will delay the healing of wounds^4^. Consequently new protocols should be used for wound healing.

Wound healing demonstrates an exceptional cellular activity process that is characteristic in wild life. However, the phase of skin repair requires the interaction of cells, growth factors, chemokines, cytokines and other signaling molecules^5–11^, which play an essential role in cutaneous injury repair. However, wound healing process involves three overlapping phases such as inflammation, tissue formation and tissue remodeling. Meanwhile, these phases should take place in order to attain tissue integrity following wounding in adult mammalian tissue^12^. As a result, several studies and many numerous technical solutions have been focusing on achieving more efficient wound treatments to minimize health costs and to provide long-term relief. By the same token, inhibiting infections and eventually successful scar healing are also targeted^13,14^.

However, many forms are used for wound dressing such as mat-fibers, hydrogels, hydrocolloids, sponges, films and membranes. Although, the fiber form surpasses the other forms wherein to mimic the extracellular of skin matrix with high porosity^15–17^. Along with these scientific advances, five different methods, including drawing, phase separation, self-assembly, template assisted synthesis, and electrospinning have been used to synthesis nanofibers scaffolds for tissue engineering. Electrospinning is the most cost-effective and simple method to fabricate a different of polymeric nanofibers^18^ whereas, the electrospun nanofiber with nano-size structure has shown many advantages when used for wound dressings due to its high porosity, very large surface area, and similarity to human cellular matrix. Furthermore, the nanofiber structure helps in absorbing wound fluid and in providing the breathability for cells proliferation and growth.

Among the biopolymers iota carrageenan (IC) is an ideal candidate for drug delivery, wound healing and tissue engineering applications^19^. However, the fiber structure of carrageenan has low mechanical properties. Therefore, a crosslinking with other synthetic polymer such as polyvinyl alcohol (PVA) needs to enhance the mechanical properties of carrageenan^20,21^. Whereas PVA is a green synthetic polymer with biodegradability, biocompatibility, chemical resistance, moisture absorbency and fibers formability^22,23^. In addition, PVA nanofibers are suitable for wound dressing applications due to their ability to transport and to absorb wound fluid and to help in the tissue regeneration because of their oxygen permeability, biocompatibility and non-toxicity^24^. For instance, Thomas et al. have accelerated the wound healing using PVA/IC hydrogel film^25^. Furthermore, Zhang et al. prepared nanofibers scaffold from electrospun PVA with graphene oxide and they realized it as a good result as being effective wound dressing^18^. While Shin et al. have proved that graphene-based materials incorporated nanofibers could promote skin regeneration^26^. Whereas, graphene-based materials have many functional groups, high surface area and protein adsorption affinity in addition to its antibacterial properties, so they were used for wound dressing to prevent infection in different wounds^26,27^. Many studies have demonstrated that small quantity of GO (< 5%) could enhance the properties of the wound dressing polymeric matrix, such as mechanical strength, protein affinity, wound healing acceleration, and the promotion of bone tissue generation^26–30^.

Therefore, prGO was introduced in this study as doping agent into PVA/IC blend to enhance its bioactivity. The morphology and the structure of this novel wound dressing (MNS) was demonstrated by using TEM, FTIR, BET and XRD and was shown for its feasibility to be used as efficient wound dressing that had been verified by in vitro and in vivo experiments.

## Results and discussion

**Figure 1:**
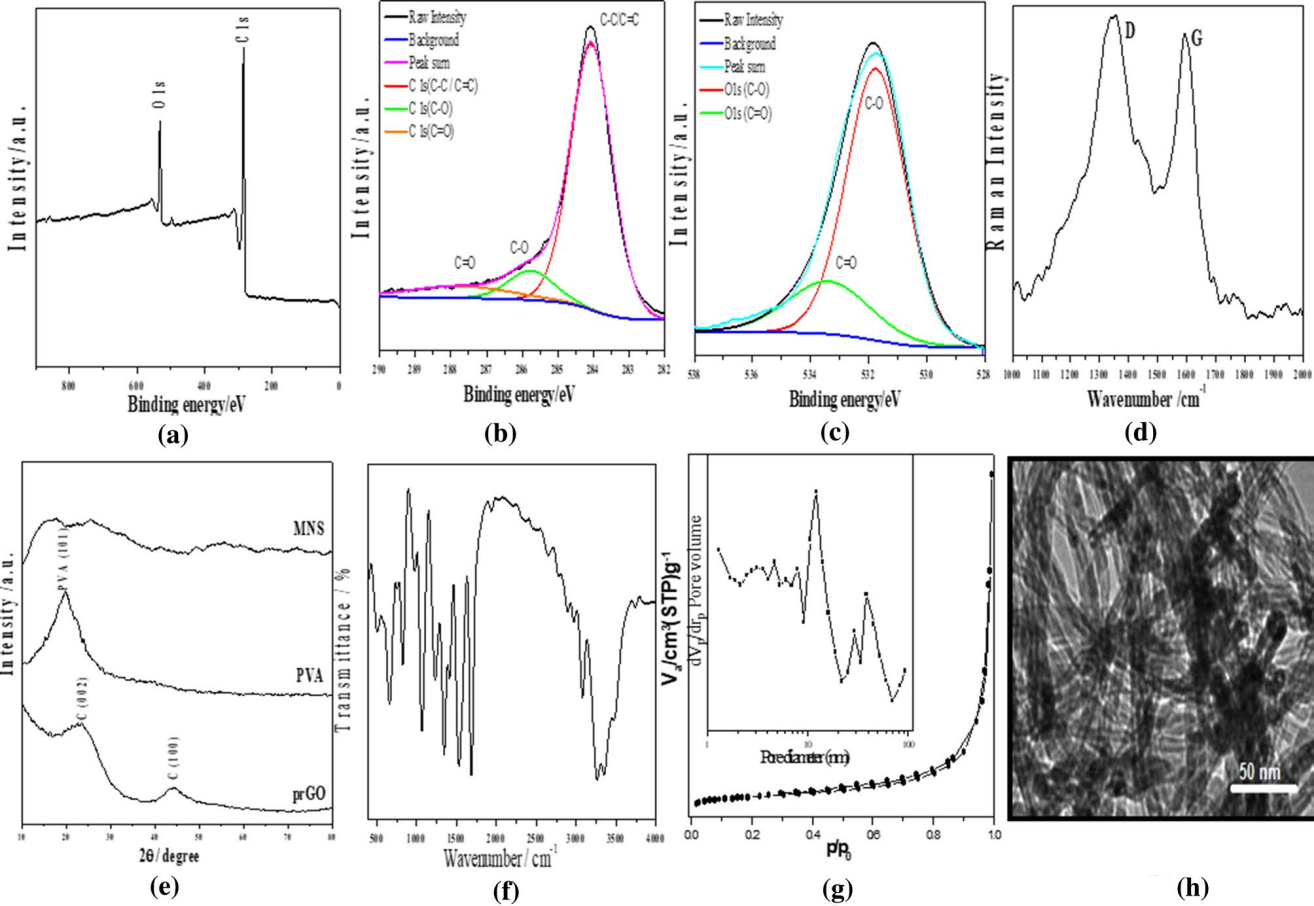
(**a**) XPS survey spectrum of prGO with (**b**) typical high-resolution XPS spectra of C1s and (**c**) typical high-resolution XPS spectra of O1s, (**d**) Raman spectrum of prGO, and (**e**) XRD spectra, (**f**) FTIR spectrum, (**g**) Nitrogen sorption isotherm with inset plot represent the BJH plot of MNS pores diameter, and (**h**) TEM image of MNS electrospun nanofiber scaffold.

The chemical states of the various elements and the presence of functional groups of prepared partially reduced graphene oxide (prGO) were explored by using XPS whereas, the scan survey spectra (Fig. 1a) had mainly showed the presence of carbon and oxygen. Figure 1b shows the high-resolution XPS spectra of C 1s region which decomposed peaks of C–C/C=C, C–O and C=O appeared at binding energies of 284, 285.8 and 287.3 eV, respectively. While Fig. 1c shows high-resolution XPS spectra of O 1s region which decomposed peaks of C–O and C=O appeared at binding energies of 531.7 and 533.4 eV, respectively. From the XPS analysis, it was concluded that, the surfaces prGO is a hydrophilic surface due to the presence of the oxygenated function groups such as carboxyl and carbonyl groups.

The Raman spectrum of prGO was explored in the range 1000–2000 cm^−1^ as shown in Fig. 1d. The D band, which was located at 1337 cm^−1^, had arisen from both the disorders and the defects in the graphitic lattice. While the G band at 1591 cm^−1^ corresponded to the Raman allowed E_2g_ optical phonon^31^. The defects in prGO structure can be quantified according to the origins of the D and G bands, and the I_D_/I_G_ ratio which is 1:1 and that defect in the prGO structure is confirmed from the XRD spectrum as shown in Fig. 1e, whereas a wide diffraction peak detected at approximately 22.6° which attributed to the (200) plane of the graphitic amorphous structure^31,32^ with d-spacing of 3.64 Å. However, by adding prGO to the polymers blend, the characteristic peak of the reflection from (101) of a monoclinic unit cell of PVA (the major matrix) at approximately 2θ of 19.92° was disappeared and an amorphous MNS structure was formed.

On the other hand, Fourier transform infrared spectroscopy was used to investigate the functional groups of the prepared MNS nanofiber scaffold as shown in Fig. 1f. The characteristic bands of IC appeared around 845, 805, 930 cm^−1^^21^. The bands between 900–1200 cm^−1^ were due to sulfonic groups of IC while the band around 1700 cm^−1^ was referred to carboxylic groups of prGO. The board bands appearing between 3078 and 3470 cm^−1^ were characteristic of the stretching –OH groups of the PVA and prGO^22^. A strong band appearing at 1650 cm^−1^ resulting from hydrogen bonds was formed between –OH groups of IC and PVA and oxygen functional groups of the prGO. A band appearing at 2200 cm^−1^ was referred to the C-H bond of the polymers in the MNS^23^.

The pore structure of MNS was examined by N_2_ adsorption/desorption isotherms, and the results are shown in Fig. 1g, whereas, it exhibits a type III adsorption isotherm on macroporous adsorbent with weak affinities. This tight desorption hysteresis was usually due to the narrow pore size distribution. In addition, the micropores were dominant in the fibers as shown in the inset of Fig. 1g, which contribute to the specific surface area as observed in TEM image in Fig. 1h. It was also noticed that, the prGO planner size was smaller than the formed MNS scaffold nanofiber diameter (as illustrated in Fig. S1 in Supplementary Materials file) and was distributed across completely wrapped on fiber shell and inside fiber core.

The physical behavior of the MNS in contact with deionized water and the tensile strength for dry sample were investigated and the results were illustrated in Table 1. The gel fraction indicates the number of crosslinking formed, which was (8%), whereas, the crosslinking process in this scaffold depended on hydrogen bonds that were formed between –OH groups of PVA, IC and prGO. Therefore, prGO is considered as bonding agent in the scaffold composite by bonding between its –COOH groups and –OH of polymers using esterification reaction. However, the swelling ratio measurement in general describes how the ability of MNS to absorb the wound exudates which will accelerate the wound healing time. On the other hand, the contact angle measurement demonstrates MNS scaffold's hydrophilic property and its ability to absorb wound liquid were compared to previous researches based on PVA^18,33–35^. Meanwhile, the mechanical strength achieved using MNS was 3MP which came in the same context with previous studies^18,33–35^ as being illustrated in Table S1.

**Table 1:** Physical properties of the MNS.

Scaffold	Thickness (µm)	GF* (%)	SR* (%)	Contact angle (°)	Tensile strength (MPa)
MNS	110	8 ± 0.2**	30 ± 0.3	40.36 ± 1.5°	3 ± 0.01

*The gel fraction (GF %) and the swelling ratio (SR %) of MNS after immersion for a day in deionized water.

**The measurements were replicated three times for the same prepared membranes and the standard deviation was evaluated accordingly for all tests.

**Figure 2:**
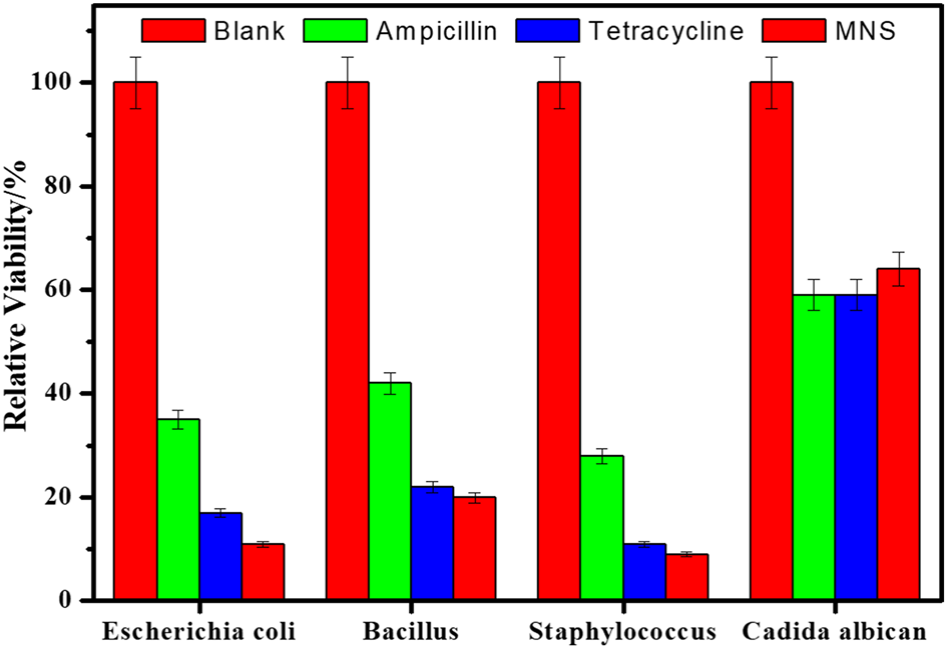
Relative viability diagram after 24 h of incubation with various antimicrobial agents against different types of microorganisms.

The results obtained from the microbial investigation represented an evidence on the compound’s inhibitory power for a stated duration. Whereas, a routine sampling was performed to assess changes over time in the viable number of cells. Relative viability after 24 h incubation of the various microbes with MNS, Tetracycline and Ampicillin were calculated and were compared with the blank (untreated microbe) as shown in Fig. 2. In addition, MNS eliminate *Aspergillus fumigatus* growth. The results reveled that the MNS has excellent antibacterial properties with different category of microbes (gram negative, gram positive, yeast and fungi) in comparison to other products^36^ and it is more effective than ampicillin and tetracycline.

The cytotoxic effect of MNS was studied by MTT evaluate whereas, the highest IC_50_ and EC_100_ values refer to the tested sample was the safest on the proliferation of normal human cells and it was found that the effective concentration (IC50) and safe dose (EC100) values were 5.095 ± 0.099* mg and 0.967 ± 0.017* mg respectively, however, the data are expressed as the mean ± SEM and (*) indicates significant difference at *p* < 0.05.The results of in-vitro cell activity testing displayed that the prepared MNS was effective in inhibiting microbial growth and had good biocompatibility and promoted cell proliferation compared to literature^37–39^. On the whole, it basically meets the requirements of an ideal antimicrobial scaffold.

**Figure 3:**
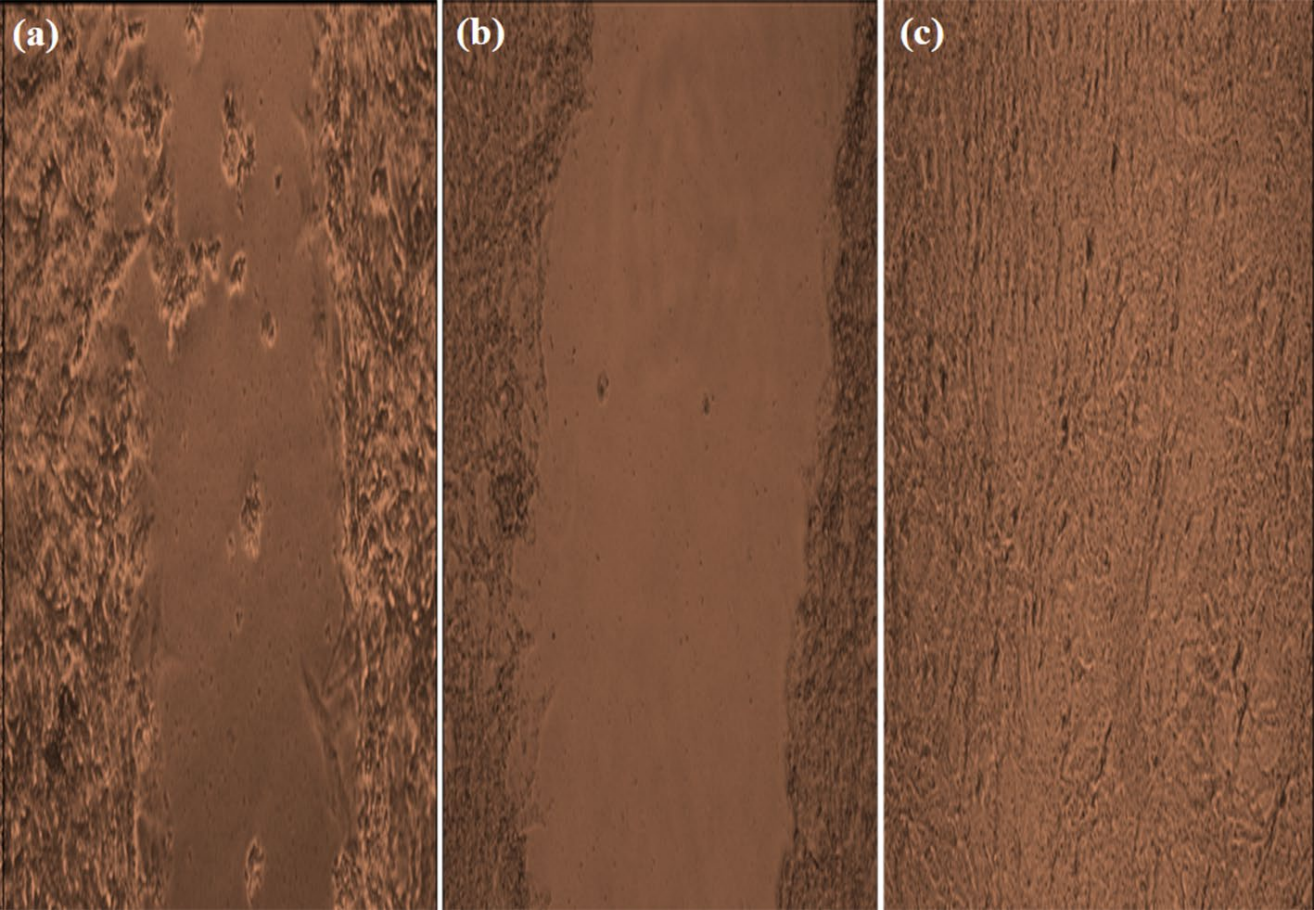
Photoimages for wound healing activities of (**a**) MNS against (**b**) the scratched Wi-38 cells in control with (**c**) unscratched Wi-38 cells (magnification × 200).

MNS was able to heal the induced wound in the scratched Wi-38 cells by 22.37%. Figure 3 shows a higher increment in the migrated cells towards the scratched area to aid in the wound closure in MNS-treated cells.

As illustrated in Table S2 in the Supplementary Information, there was a significant difference among various biochemical parameters between the different treated groups (groups 3, 4, 7 and 8) and between the untreated ones (groups 1, 2, 5 and 6) with MNS in which the presence of the synthetic MNS increased the total protein levels and decreased LDH which was released in cases of tissue damage to pinpoint an indication to common injuries. Lactate dehydrogenase (LDH) is a lactic acid dehydrogenase enzyme present in almost all body tissues. It is necessary for cellular respiration, that is the mechanism by which glucose (sugar) from food is transformed into energy for our cells^40,41^. Total proteins are important building blocks for all cells and tissues. They are important for body growth, development, and health. They form the structural part of most organs and make up enzymes and hormones that regulate body functions. This test measures the amount of protein in the blood. The liver produces albumin, which accounts for about 60% of total protein. Albumin nourishes cells, prevents liquids from flowing out of blood vessels, and transports hormones, enzymes, medications, and calcium throughout the body. Albumin, a non-plasma specific enzyme released from the sinusoidal surface of the liver cell, is found in the serum at low levels in the absence of liver fibrosis^42^. This indicates the ability of the prepared MNS to decrease tissue damage. Furthermore, there was no significant difference in creatinine between all the groups as confirming as there was no effect on the kidney functions. In addition, GGT, ALP, ACP, ALT and AST, which are strong indicators of liver functions, showed no significant variation for indicating the lack of hepatotoxicity among various groups receiving different treatments.

Since the normal control rats were essentially normal, the hematological values of the normal control rats were used as the reference values. In addition, the MNS-treated rats had values that were similar to those of the usual control rats. While white blood cells (WBCs) or leukocytes are much less than red blood cells, they play an essential part in the body’s disease defense. There are 4000 to 11.000 WBCs/mm^3^ on average, and they make up less than 1% of overall blood volume^43^. For WBCs, Lymph#, Mon#, Gran#, Lymph, Mon, Gran, RBCs, HGB, HCT, MCV, MCH, MCHC, and RDW, there was a substantial gap between the MNS-treated and the non-treated negative controls as being confirmed from the illustrated data in Table S3 shown in the Supplementary Information.

The infected groups by *E. coli* and *Staphylococcus* showed a significant increase in WBCs, however, using synthetic wound dressing in addition to yeast in drinking water had alleviated such increase i.e. decreased inflammation. These results are collinear with the previous work^44^ which emphasized the anti-inflammatory effects of a hydrogel containing polyvinyl alcohol (PVA) in a ratio 10%, in addition to the impermeability of PVA to bacteria^45^. Consequently, their gels reduce the primary wound contamination as well as their ability to protect the wound from secondary bacterial infection^46^.

**Figure 4:**
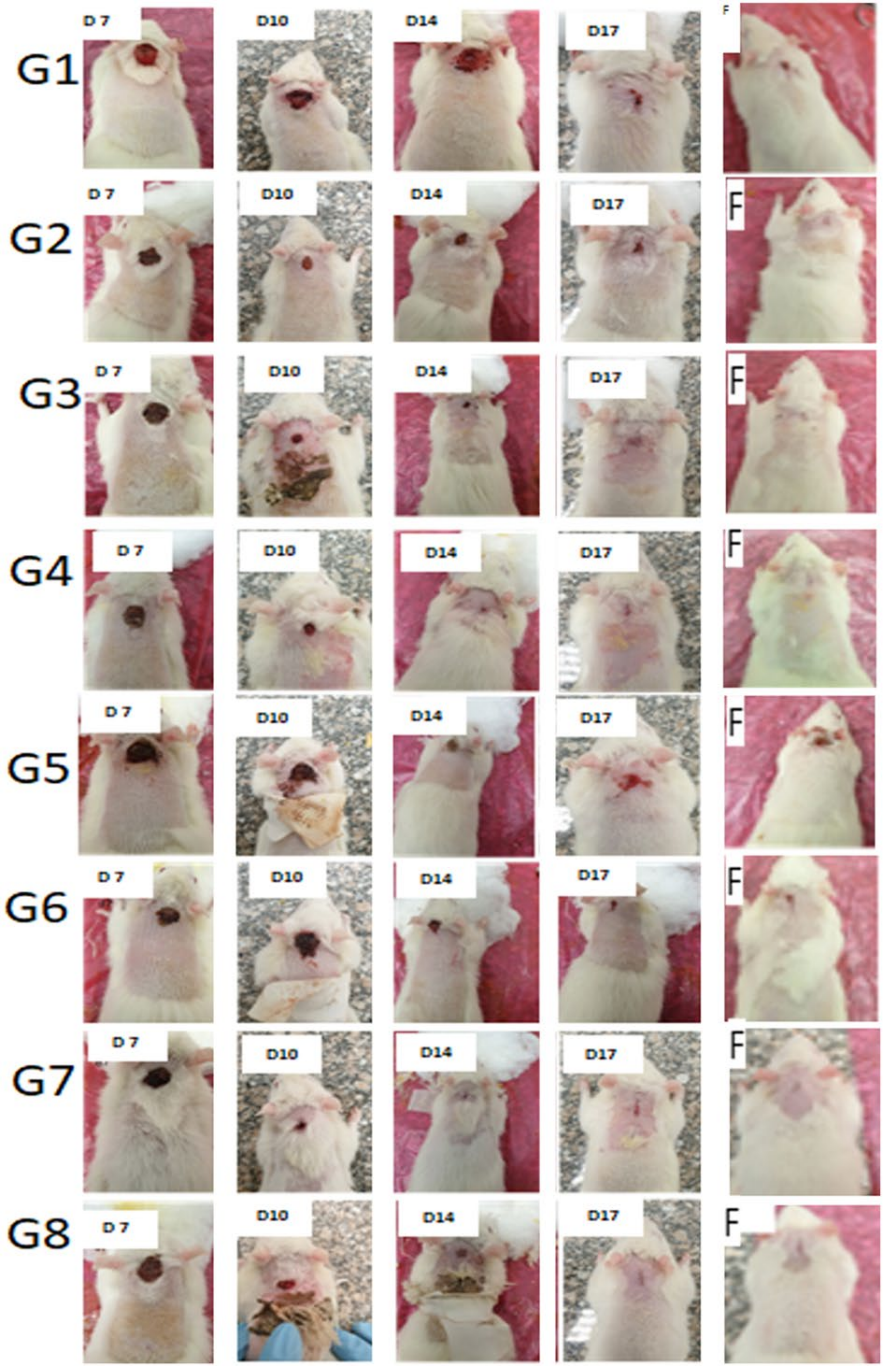
Photoimages for wound treatment to the different groups at days 7, 10, 14, 17 and 28.

Images of wound were taken for the different groups at days 7, 10, 14, 17 and 21 as shown in Fig. 4. They expressed the ability of the synthetic MNS used in groups 3, 4, 7 and 8 to improve wound healing process. The presence of yeast as a natural supplement improved the hair growth in both cases of normal and infected wounds in groups 4 and 8 respectively. These results were confirmed by histopathological findings. However, the wound healing process took place after 21 days of complete healing with the decomposition of the used scaffold, and this was a great benefit, whereas it can be used as surgical sutures for the inner layers of the body for its complete decomposition. Also it can be used in cosmetic surgeries due to the disappearance of surgical effects after using this scaffold.

**Figure 5:**
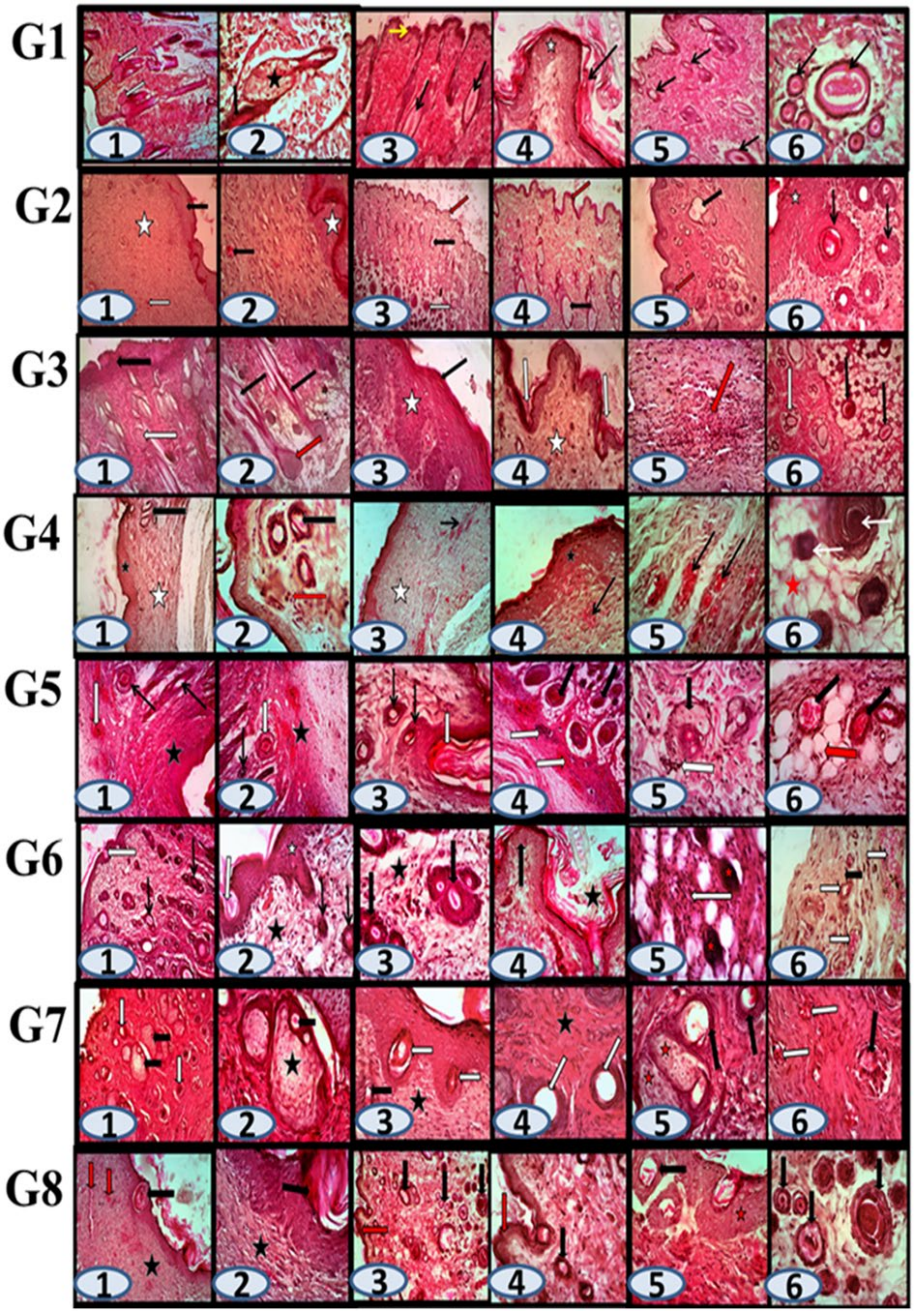
Histological view of skin wound for all studied groups stained with hematoxylin and eosin (H&E).

Figure 5 shows the histological view of skin wound for different groups (in brief, the figure’s details has been illustrated in the Supplementary File), whereas the microscopic examination for the healed skin wound in the control group 1 had revealed the presence of almost normal thickness of the epidermal layer, with dermal constituents of hair follicles and associating active sebaceous glands in a derma fibrous connective tissue elements. The surface of the epidermal epithelium at the site of healing appeared irregular or wrinkled and it was covered with excess keratin sheets of stratum corium. The present hair follicles especially in deep dermis appeared as numerous and as of variable size.

Comparatively, the microscopic changes in healed wound in group 2 treated with gentamicin, revealed the presence of an area of nearly thickened epithelium(black arrow), wide dermal connective tissue (asterisk) and various vasculatures (white arrows),the dermis contained excessive fibrous connective tissue proliferation and small congested blood capillaries. Other cases showed an active regeneration with a corrugated epidermal epithelium (red arrow) in addition to an excess of variable sized hair follicles that appeared dilated and mat cystic with intra-luminal pale stained hair and keratin contents.

The case of group 3 which was treated with synthetic MNShad shown small region of im-proper complete healing, where the epidermal epithelium appeared thickened and with the presence of some foci of coagulative necrosis (black arrow in image 1). The dermal connective tissue proliferation was accompanied with the presence of numerous hair follicles and with enlarged sebaceous glands (white arrow), especially, at the bulge end of some hair follicles. The thickened epidermis with hyperplastic prickle (asterisk) and keratinocytic cell (black arrow) had been accompanied with the appearance of numerous dermal papillae into the epidermis. It was noticed that the keratinization as well as the dermal mononuclear inflammatory cells were numerous (red arrow in image 5). Excess cross sections in hair follicles were seen at the deep dermis as shown in (image 6) whereas upper hair follicles (white arrow) and other deep hair follicles (black arrows) had appeared as surrounded by excess adipocytes.

However, the microscopy for the healed wound in rats of group 4 revealed presences of a wide area of fibrous connective tissue covered with somewhat thick layer of re-epithelialization, while few cross sections of hair follicles were still present. Most of the present blood vessels all over the dermal connective tissue appeared congested. On the other hand, the microscopy images for group 5 (the control group for bacterial infection) revealed the presence of coagulate necrosis as well as liquefaction of most of epidermal epithelium and dermal layer. In these areas excess of mononuclear and poly-morpho-nuclear inflammatory cell were present. Some few present hair follicles appeared degenerated and associated with less numbers of sebocytes. Vascular changes of congestion and hemorrhages were also observed all over the dermis and hypodermis.

The healing of infected wounds that were treated with gentamicin (group 6) appeared with obvious proper microscopic changes than in the only infected wounds. Nearly normal somewhat thicker epidermal epithelium with keratinization was seen. Numerous of nearly normal hair follicles of variable sized, were present as some of which appeared dilated and associated with a variable numbers of mononuclear inflammatory cell infiltration. The blood vasculatures all over the dermis and the hypodermis appeared congested with less hemorrhages.

However, the microscopic images of group 7 (*E. coli* and *Staphylococcus* infected wound and dressed with MNS) revealed degeneration and atrophy and were smaller in size of the present hair follicles (arrows) and sometimes dilated with nearly no hair. In the meantime an obvious hyperplasia in the sebocytes and sebaceous glands (asterisk) encircling the hair follicles, was noticeable and had led to the atrophy of some follicles. Slight changes of vascular congestion and inflammatory cells were observed in image 6 whereas dermal fibrous connective tissue elements with congested blood capillaries (white arrows) and one damaged hair follicle had appeared.

The microscopy for wound healing for the rats in group 8 revealed main changes of re-epithelialization epidermal thickening, dermal fibrous connective tissue proliferation and slight vascular congestion whereas enlarged epidermal in fold (image 1) contained excessive compact keratin (black arrow) and congested vasculatures (red arrows) in the dermis (asterisk). However the higher magnification (image 2) showed the hyper-keratinization in the epidermal infold (arrow) and under fibrous connective tissue of the dermis (asterisk) . The wall of some follicular ductules (arrow) appeared thick (image 5) from epithelialization(asterisk).While, excess of variable sized hair follicles (arrows) in the deep dermal loose connective tissue has been shown in image 6.

Rather than the basic role for the healing of wounded skin, that mainly depends on (dermal fibrous tissue proliferation with its constituents of hair follicle and sebaceous gland, followed by epithelial regeneration either epidermal or follicular), it becomes somewhat variable according to the used methods for treatments. In the present study, nearly a normal role was observed in group 1 for the healthy non infected and un-treated wound when having considerable skin epidermal and dermal parameters that were nearly regenerated. After the treatment with gentamicin in group 2, changes were progressed by the excess of dermal fibrous connective tissue and epidermal re-epithelialization, while the hair follicles and sebaceous glands were somewhat active. On other hand the use of MNS in the case of group 3 led to appearance of small foci of epidermal necrosis, some other areas of the epidermal epithelium were multiple and thickened with observable numerous dermal papillae. Some mononuclear cells were seen among the proliferated dermal connective tissue, while the hair follicles were excessive and sebaceous glands appeared active, especially, at the bulge end of the follicles that had increased for 4th group where the wounded skin that was treated with the artificial sheet and yeast. Considering the groups of *E. coli* and *Staphylococcus* that had infected wounded skin, some other advanced changes were seen. The 5th group that only had infected and non-treated wounds, severe changes of necrosis with liquefaction besides excess inflammatory cells, congestion and hemorrhages plus clusters of microorganisms were seen at the upper layers of the wounded skin. The present hair follicles and the sebaceous glands had been degenerated and had become necrotic except for those at the lower dermis and the hypodermis. The vascular congestion was still clearly observed at the deep blood capillaries and venules. On the other hand, the 6th group with infected wounded skin that was treated with gentamicin had shown better healing repair than the other 5th group. The superficial epithelium was regenerated and became thick. An excess of the underlying dermal contents of newly formed hair follicles, fibrous tissue proliferations, congested blood vessels and some mononuclear cells were observed. No obvious or active sebaceous glands could be detected and seen in this group. The other group with the infected wound treated with MNS (group 7) was characterized by an obvious thicker and excessive epithelial regeneration of the epidermis. In addition, the wall of the ductules of hair follicles having more active and hyperplastic sebaceous glands, few degenerated hair follicles may be seen. The healing in the last group with infected wound was that treated by MNS and by yeast (group 8), was similar in the observable results. In this group the healing mainly depended on the dermal fibrous connective tissue proliferation with thick, corrugated and keratinized covering regenerated epidermal epithelium. The present deep hair follicles were degenerated and were accompanied by some congested blood vessels. In conclusion, the ideal scaffold should have broad-spectrum bactericidal activity with appropriate mechanical properties in addition to its ability to wound healing^15–17,37–39^.

There was no change in the expression of interleukin (IL1 & IL6) genes among different groups although they were slightly increased in the case of the group that took yeast. The REG (regenerating protein) I alpha promoter activity in individually cells was substantially enhanced by IL-6 stimulation. The anti-inflammatory cytokine IL was produced to prevent the excessive inflammation during the healing of a skin wound^47^. Also, there was definitely no dissimilarity in the expression of cyclooxygenase (COX) gene among the diverse groups. However, COX enzymes play an impotent role in the transformation of arachidonate to (Prostaglandin H2) PGH2^48^.

**Figure 6:**
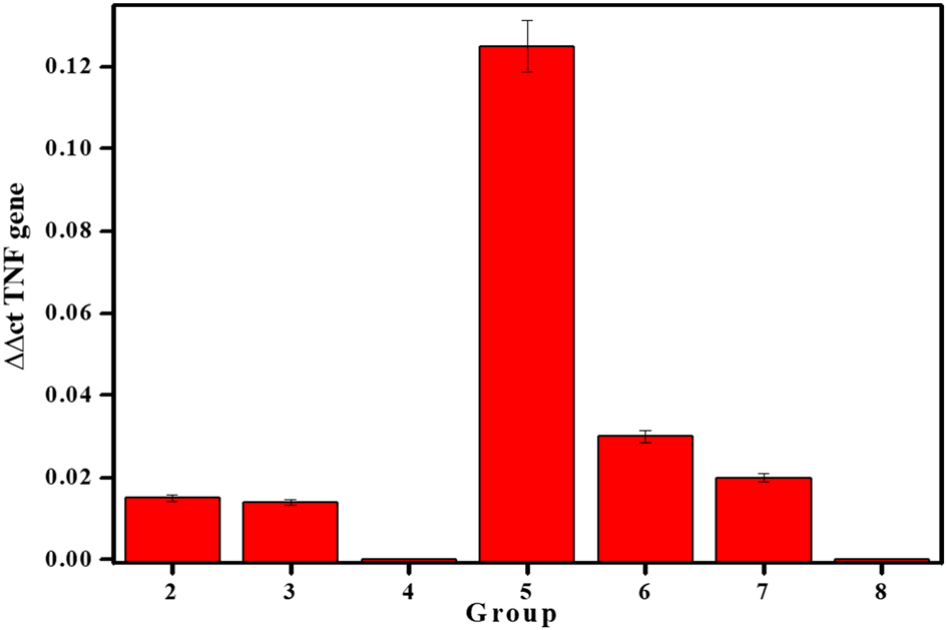
Genes expression of TNF of studied groups during the experimental period.

The data illustrated in Fig. 6 showed genes expression of TNF for different groups under the study during the experimental period and the results reveled that yeast had regulated TNF gene expression with folding greater than that without yeast as being presented in group 4 and group 8.

In order to explain the possible achievement mode of the treatments at the molecular degree of TNF (pro-inflammatory cytokines) gene expression, the expression levels in group 5 was higher than that all of the groups under investigation due to presence of contamination which increase the inflammation. On the contrary, group 4 and group 8 had no variation in the TNF gene expression, and that may be due to, the increase in the expression of TNF (proinflammatory cytokines) gene during the inflammatory and the late proliferative phases formation during the wound healing processes that had encouraged proteins to involve in tissue repair^49^. All the aforementioned events for the differential gene expression had been well correlated with distinct gene families typically identified by gene cluster analysis. These genes included cellular and intercellular mediators and their proteinases. The creation of restorative mechanisms such as growth regulatory genes and genes encoding for the production of structural proteins, occurred at later time^50^.

Furthermore, using the gentamycin treatment or MNS, the expression of the TNF gene was identical and was closest to normal cell expression. Thus it can be concluded that the prepared MNS becomes usable as being a protective shield.

## Conclusion

In this study a novel nanofibers scaffold composed of PVA/IC/ GO nanocomposite (MNS) was successfully prepared by an electrospinning method and an improvement in wound healing of Wi-38 cells by 22.37% was observed as well. Such findings were confirmed by the obtained results from in vivo study in which it improved wound healing process in rats and in the presence of yeast as a nutritional supplement induced hair growth in the site of injury for both cases of the infected and non-infected wounds. Consequently, preventing the formation of scars in these regions, MNS is also effective as an antimicrobial agent. In conclusion, the ability of MNS with its eco-friendly, its availability and its safe components can be used as an effective wound healing dressing.

## Experimental

### MNS scaffold fabrication

Partially reduced graphene oxide (prGO) was prepared from PET bottle waste as mentioned in a previous work^51,52^. The resultant black powder was collected and crushed using ball mill. For scaffold fabrication, at the beginning 10 g of PVA (99% hydrolysis and medium MW,USA) was dissolved in 100 mL deionized H_2_O at 90 °C for 2 h and 1 g of IC (type V) was dissolved in 100 mL deionized H_2_O at 80 °C for 1 h then blending PVA:IC:GO (95:3:2) by weight ratio respectively. After that the electrospinning of polymeric composite was made at voltage of 20 kV, and distance between tip of syringe and stationary collector was 15 cm where nanofiber composite was collected on the stationary collector.

### Antimicrobial properties

*Escherichia coli* (garm negative bacteria) and *Bacillus & Staphylococcus* (gram positive bacteria), *Candidaalbican* (yeast) and *Aspergillus fumigatus* (fungus) were used for the detection of antimicrobial studies. All microorganism were inoculated into the LB liquid medium under 200 rpm and 37 °C, until the OD value at around 600 nm was about 0.6. The bacterial solution was diluted at a volume ratio of 1:1000.Sterilized MNS (0.01 g) was added to the 3 mL bacterial solution and incubated at 200 rpm, 37 °C, for 24 h. The value of the OD at 600 nm has been calculated and compared to the same concentration of antibiotics.

### Biocompatibility

The Human Normal Fetal Lung Cell Line (Wi-38) was used according to the method defined by Mosmann, 1983^53^ to investigate toxicity. Human Wi-38 cells with 10 percent fetal bovine serum were preserved in DMEM medium (ATCC “the American Type Culture Collection”, Lonza, USA). For 2 weeks before assay using trypsin EDTA, this cell line was subcultured (Lonza, USA).Their viability and counting were detected by trypan blue stain and hemocytometer. Wi-38 cells were seeded in 24 well culture plates as 5 × 10^4^ cells per well and incubated at 37 °C in 5% CO_2_ incubator. After 24 h., cells were treated with 1 mg of MNS. MTT solution (Sigma, USA) (5 mg mL^−1^) was additional to individually well next 72 h at 5% of CO_2_ incubator, and 37 °C for 4 h in 5% of CO_2_ incubator.MTT solution was removed and 100 percent DMSO 150 μL was added.The absorption of each well was measured at 570 nm using a microplate reader (BMG LabTech, Germany) to estimate the percentage of cell viability after MNS exposure compared with the untreated cells. The effective concentration (IC_50_) and safe dose (EC_100_) values of the tested compounds that cause 50% and 100% cell viability were estimated by the Graphpad Instate software. Data are expressed as the mean ± SEM.

### In vitro study

Assay for wound healing was performed as described by Liang et al*.*^54^ with some modifications. Wi-38 cells were planted in 24 well cell culture plates at a concentration of 5 × 10^4^ cells. The cells were incubated for 24 h to form confluent cell monolayer. A small area was then scratched using sterile pipette tip. After washing, safe dose (EC_100_) of MNS was incubated with cells for 24 h. in 5% CO_2_ incubator. The cells which migrate to fill the scratched area were captured by digital camera attached to phase-contrast inverted microscope with digital image analysis system (Cellsens Soft Imaging System).Moreover, these cells were measured by MTT examine to estimate the measurement of wound healing after treatment with MNS.

### In vivo study

Forty Male Wistarrats weighing 150–200 g were used for this study. The rats were purchased from Medical Research Institute, Alexandria University, Egypt. The animals were allowed to acclimatize for one week prior to the experiment. The rats were preserved in clean polypropylene cages through stainless steel top grill under standard laboratory conditions (25 ± 2 °C), humidity (60 ± 10%) and 12 h light/dark cycle, and were fed with standard pellets diet and water ad libitum in clean polypropylene flasks with stainless steel sipper tubes. There was regular washing of the cage bedding and water bottles. Rats were randomly assigned into eightexperimental groups of 5 animals each (n = 5 per group). The grouping of animals was done as follows: Group 1 served as untreated control group; Group 2: rats that dressed with gentamicin ointment as a reference standard; Group 3 was treated with synthesized wound dressing; Group 4 was treated also with synthesized wound dressing. In addition the administration of yeast in drinking water (5 mg kg^−1^ BW).Whereas, Group 5: Infected with bacteria (*E. coli* and *Staphylococcus*) were being left untreated; Group 6: Infected with bacteria and dressed with gentamicin ointment; Group 7: Infected with bacteria and treated with synthesized wound dressing; Group 8: Infected with bacteria and treated also with synthesized wound dressing in addition to administration of yeast in drinking water (5 mg kg^−1^ BW). All groups were treated for 21 days. Wound healing activities were evaluated using excision wound model. After overnight fasting, all the rats were anesthetized with ketamine hydrochloride (100 mg kg^−1^). For all the groups, the dorsum of the rats was shaved with an electric clipper and cleaned with povidine iodine and 70% alcohol. A sterile towel was used to isolate the operation site. Excision wound was created. Anaesthetized animals were placed on the operation table in its natural position. Using toothed forceps, scalpel and pointing knives, a full-thickness wound of around 1 cm diameter and 0.2 cm depth was made on the dorsal thoracic region of rats. The haemostasis was obtained by smearing the wound with a swab submerged in saline solution. The wounds were then dressed on the 3rd, 7th, 10th, 14th, 17th and 21th days post wounding as per the experimental protocol and evaluated for healing on these specific days considering the wounding day as Day “0” till the epithelialization was completed. In addition to the size reduction and percentage of wound closure, the wound healing and closure rate was measured by tracing improvements in the wound area, the time of epithelialization and the number of days needed for the collapse of dead tissue fragments lacking any remaining fresh wound. To avoid the loss of dressings during the movement of the rats, perforated gauze cloths were used to hold the dressings in place. Wounds were traced with the help of digital photographs that were taken.

### Blood tests

Blood samples were collected from the sacrificed animals and centrifuged at 3000 rpm for 20 min to find plasma. Plasma samples were stored at − 80 °C were analyzed for total protein, albumin, creatinine, GGT (gamma glutamyl transferase), ALP (alkaline phosphatase), ACP (acid phosphatase), AST (aspartate transaminase), ALT (alanine transaminase) and LDH (lactate dehydrogenase) using kits purchased from BioSystems S.A. Costa Brava, 30. 08030 Barcelona (Spain). Heparin was used as an anticoagulant in plasma samples and non-coagulated blood was tested, for WBCs, Lymphocytes(Lymph), Monocytes (Mon), Granulocytes (Gran), Lymph %, Mon %, Gran%, RBCs, HGB, HCT, MCV, MCH, MCHC, RDW % by Mindray hematology analyzer.

Plasma samples stored at − 80 °C were analyzed for total protein, albumin, urea and LDH (lactate dehydrogenase) using kits purchased from BioSystems S.A. Costa Brava, 30. 08030 Barcelona (Spain). Heparin was used as an anticoagulant in plasma samples and non-coagulated blood was tested, for WBCs, Lymphocytes(Lymph), Monocytes (Mon), Granulocytes (Gran), Lymph %, Mon %, Gran%, RBCs, HGB, HCT, MCV, MCH, MCHC, RDW % by using Mindray hematology analyzer.

### Histological examination

Skin specimens were collected from the skin at the site of application of the disparate groups of treatment. Specimens fixed in 10% neutral buffered Formalin solution. Cleared in xylene and then embedded in paraffin wax for a minimum of 24 h after the steps of dehydration in the ascending grades of ethanol. According to Bancroft and Stevens^55^, tissue parts (5–7 microns thick) were cut and stained with hematoxylin and eosin (H&E) and subjected to histopathological assessment by light microscopy.

### Molecular effect

Total RNA was isolated using RNA extraction Kit (QIAGEN). The extracted RNAs from different blood samples were quantified and qualified (purity) using NanoDrop Spectrophotometer. Finally, total RNAs samples were normalized (all samples had the same concentration) to avoid any false increase in gene expression levels. Using TOPrealTMqRT-PCR 1-step Kit (SYBER Green with low ROX, Korea), gene expression of interleukin (IL1b, IL1R1, IL6) and COX (target genes) and β-actin (reference gene) were quantified by Real-Time PCR System (CFX, BIO RAD) with the use of specific primers sequences as shown in Table S4 in supplementary information. qRT-PCR was implemented in a reaction mixture of 20 μL. qRT-PCR program was applied as one cycle at 50 °C for 10 min., one cycle at 95 °C for 5 min. and followed by 35 cycles of denaturation at 95 °C for 10 s, annealing at 60 °C for 20 s and extension at 72 °C for 30 s.

### Ethical approval

All methods were performed in accordance with the relevant guidelines and regulations, and the experiments done on animals are in accordance with arrive guidelines.

The experimental design was accepted by the local research ethical committee (REC) of experimental animal use at the City of Scientific Research and Technology Applications (SRTA-City), Alexandria, Egypt by applying the principles of replacement, reduction and refinement (the 3Rs).

## Supplementary Information

Supplementary Information.
