# Supplementary material for: Novel scaffold based graphene oxide doped electrospun iota carrageenan/polyvinyl alcohol for wound healing and pathogen reduction: in-vitro and in-vivo study
Source: Sci Rep. 2021 Oct 14;11:20456. doi: 10.1038/s41598-021-00069-0 (PMC8516857; doi:10.1038/s41598-021-00069-0)
Supplement: Supplementary file 1 — Supplementary Information. [file 41598_2021_69_MOESM1_ESM.pdf]

Supplementary Material

Novel scaffold based graphene oxide doped electrospun iota carrageenan/polyvinyl alcohol for wound healing and pathogen reduction: in-vitro and in-vivo study

Marwa H. Gouda[1], Safa M. Ali[2], Sarah Samir Othman[3], Samia Abd Allah Abdal- Aziz[2], Marwa M. Abu-Serie[4], Noha A. Elsokary[5], Noha A. El Essawy[6]*

[1]Polymer Materials Research Department, Advanced Technology and New Materials Research Institute, City of Scientific Research and Technological Applications (SRTA-City), New Borg El-Arab City, 21934 Alexandria, Egypt.

[2]Nucleic Acid research department, Genetic engineering and biotechnology research institute, City of Scientific Research and Technological Applications (SRTA-City), Alexandria, Egypt.

[3]Pharmaceutical Bioproducts research department, Genetic engineering and biotechnology research institute, City of Scientific Research and Technological Applications (SRTA-City), Alexandria, Egypt.

[4]Department of Medical Biotechnology, Genetic Engineering, and Biotechnology Research Institute, City of Scientific Research and Technological Applications (SRTA-City), Alexandria, Egypt.

[5] Pharmaceutical and Fermentation industries Development centre, City of Scientific Research and Technological Applications (SRTA-City), Alexandria, Egypt.

[6]Central labs, City of Scientific Research and Technological Applications (SRTA-City), Alexandria, Egypt.

Corresponding author: nony_essawy@yahoo.com (Noha A.Elessawy)

MNS scaffold physical characterization

X-ray photoelectron spectroscopy (XPS) with a Phi 5300 ESCA system (Perkin-Elmer) using Mg (K) radiation (1253.6 eV) was utalized to invastigate the elemental contamet of prGO in addition to, Raman spectroscopy which conducted through a Senterra instrument, (Bruker) with a 514.5 nm excitation wavelength in the range of wave numbers from 1000 to 2000 cm^{-1}. While,The crystallographic phase of the produced scaffold,prGO and rawPVA was determined by X-ray powder diffraction (XRD, Shimadzu-7000, Japan). The MNS scaffold morpholgies were identified using a transmission electron microscope (TEM, JEM-2100 plus) and scanning electron microscopy (SEM,SU-70, Hitachi, Japan). A Bruker ALFA spectrometer was used to perform the Fourier transform infrared (FTIR) study (Bruker Corporation, Germany) in a range of 400–4000 cm^{-1}. Scaffold surface area and pore volume were measured using the Brunauer–Emmett–Teller (BET) technique,with Barret–Joyner–Halenda (BJH) adsorption to invastigate the pores diameter majorty.

Gel fraction (GF%) was measured for pieces of dry MNS scaffold has weight (W_o). Then, it was soaked in H_2O for 24 h. The scaffold was dried again at 50 °C in an oven and weighed (W_e). The gelation percentage (%) was calculated according to equation (1)

$$GF\ \% = We/Wo\ X\ 100 \qquad (1)$$

While the swelling ratio (SR%) was calculated according to equation (2)

$$SR\ \% = Ws/Wo\ X\ 100 \qquad (2)$$

where W_o and W_s are weights of MNS scaffold before and after swelling, respectively.

Contact angles were measured to evaluate the hydrophilicity of the scaffold. Therefore, a contact-angle analyzer (Rame-Hart Instrument Co. model 500-FI) was used. While, the tensile strength was performed for the dry sample at room temperature by using Lloyd Instruments LR10k

until the MNS scaffold breaking. The sample dimensions were 70 mm length, 10 mm width and 80 µm thickness. The tensile rate was 5 mm/min and the test was repeated three times.

In vitro degradation test

The degradation tests were performed with three MNS electrospun nanofibrous scaffolds (1cm x 1cm) in phosphate-buffered saline (pH.4, containing 200 mg/L NaN_3 as a biocide). Each scaffold were sterilized under UV irradiation for 2 h, vacuum-dried for 24 hours, individually weighed, placed into its own sealed vial of 10 mL PBS and incubated at 37 °C.At the time of extraction, samples (3 replicates) were removed from the incubator, gently washed with deionized water to remove latent PBS salts, vacuum-dried at 40°C for 48 hours and weighed. Weight loss percentages were calculated from the dried weight obtained before and after degradation. The percentage of weight loss was determined after drying the samples in vacuum by comparing dry weight, W_d at a specific time with the initial weight, W_o according to equation (3):

$$\text{Weight losses } \%= (W_o - W_d / W_o) \times 100\% \qquad (3)$$

A complete degradation were took place after 21 days and this is a great benefit, whereas it can be used as surgical sutures for the inner layers of the body for its complete decomposition. In addition, it can be used in cosmetic surgeries due to the disappearance of surgical effects after using this scaffold.

Statistical analysis

The mechanical testing (tensile strength), contact angle, swelling ratio, gel fraction and resultant scaffold weight loss values were presented as mean values ± the standard deviation (SD) of the mean. The differences between experimental groups were examined using IBM SPSS Statistics. The data

obtained from in vitro and in vivo studies were analyzed and compared using Microsoft Excel and the mean standard error of the mean (SEM). Statistical significance was defined as a value of $p < 0.05$.

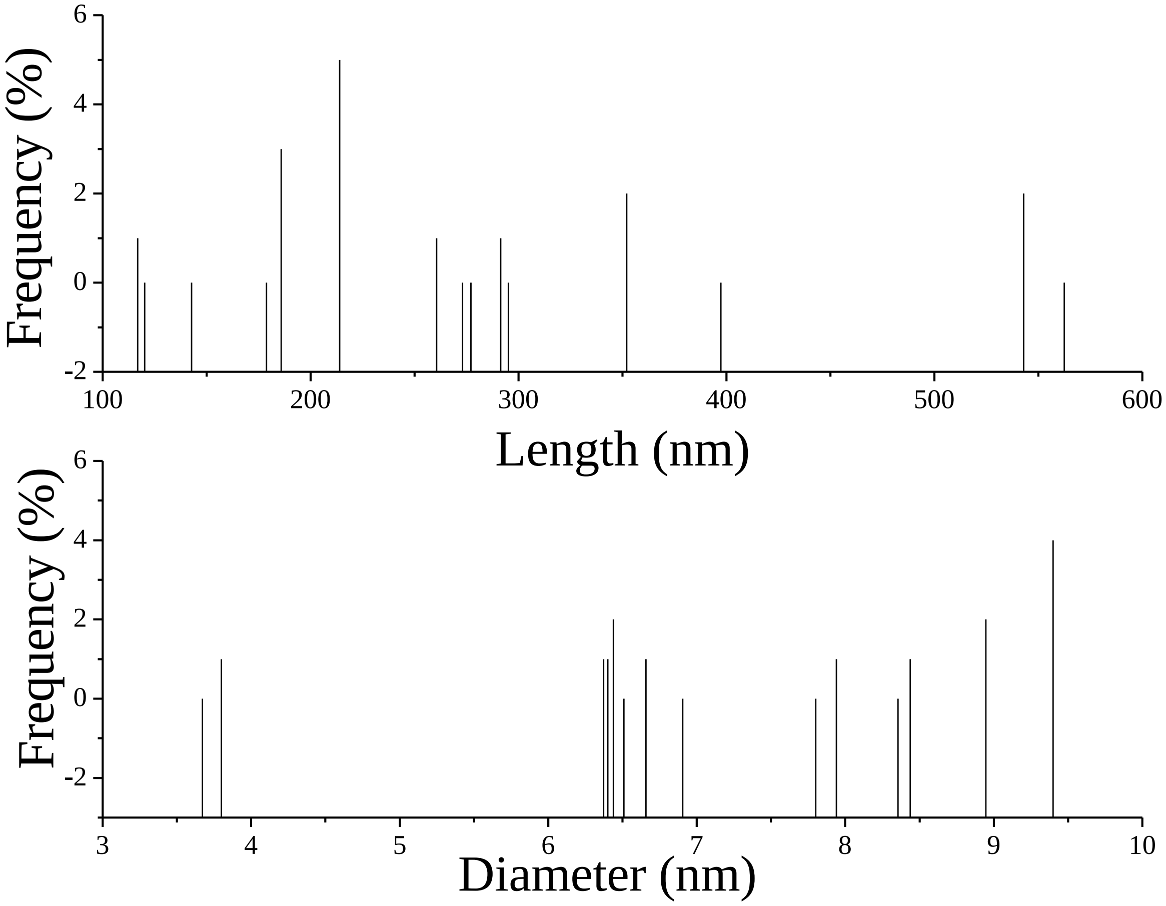

Figure S1: The frequency distribution size plot from TEM image.

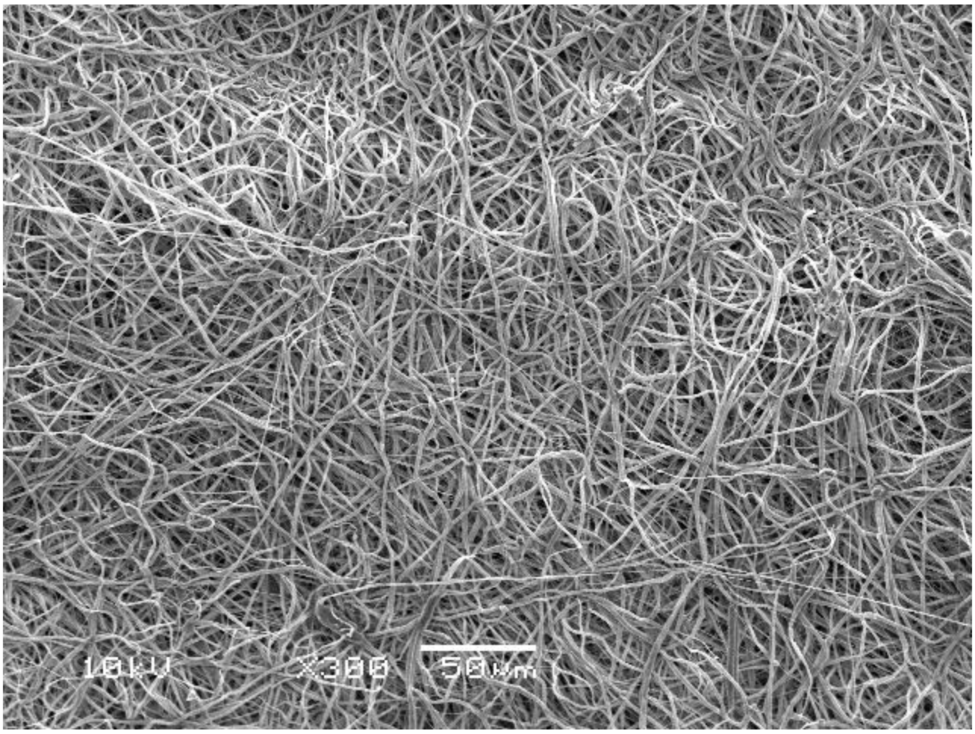

Figure S2: SEM image of MNS electrospun nanofiber scaffold.

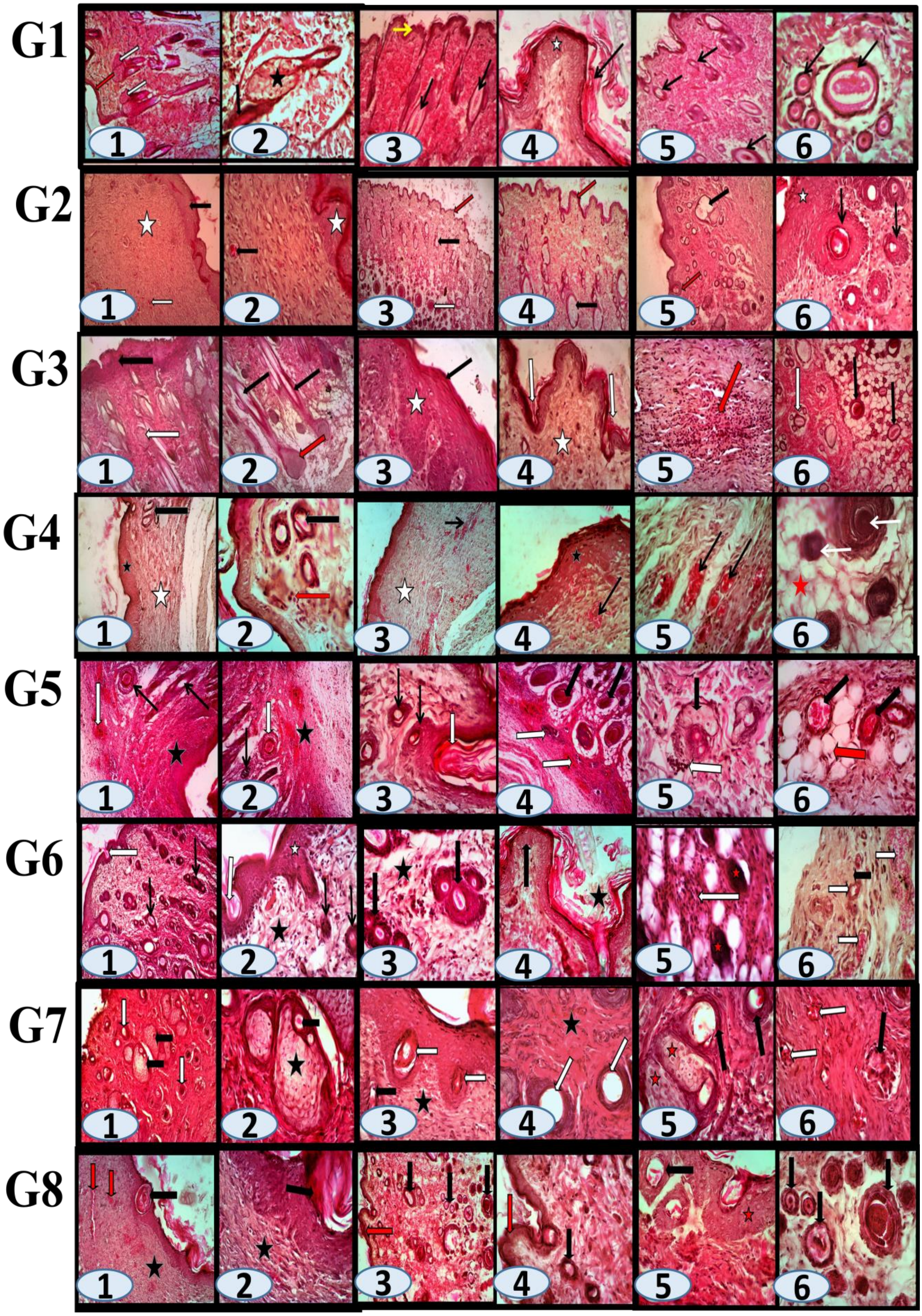
G1
G2
G3
G4
G5
G6
G7
G8
1
2
3
4
5
6

Rat dorsal skin of Gp1(1) Nearly normal epidermal epithelial layer (red arrow) with underlying hair follicles and somewhat enlarged sebaceous glands (white arrows)[H & E, X 160],(2) Higher magnification to show the hair follicle (arrow) with attached sebaceous gland (asterisk) within surrounding dermal connective tissue elements[H & E, X 400], (3) Normal skin epidermis (yellow arrow) with irregular superficial epithelium by the hair and hair follicles[H & E, X 250], (4) Higher magnification to show the active epidermal layers of epithelium (white asterisk) with covering sheets of keratin (arrow)[H & E, X 250], (5) Other view showing normal skin layers with clear dermal contents of hair follicles and follicles (arrows)[H & E, X 160], (6)Higher magnification to show cross sections in a variable sized hair follicular follicles (arrows) with central hair contents[H & E, X 400].

Rat dorsal skin of Gp2 (1) Nearly thick epidermal epithelial layer (black arrow), wide dermal connective tissue (asterisk) and various vasculatures (white arrows)[H & E, X 160],

(2) Higher magnification to show the nearly thick epithelial layer (asterisk), dermal connective tissue elements and blood capillary (black arrow)[H & E, X 400], (3) Dorsal skin with active hyperplastic corrigated covering epithelium (red arrow), dilated and cystic hair follicles (black arrow) and other excess deep variable sized hair follicles (white arrow)[H & E, X 160],(4) Dorsal skin with active hyperplastic corrigated epidermal epithelium (red arrow), and some dilatation-ectatic hair follicles with intra-luminal pale keratin (Black arrow)[H & E, X 250], (5) Nearly thick epidermal epithelial layer with embedded folds of hair follicles (red arrow), and other increased numbers of dermal follicular units, some of which are cystic (black arrow)[H & E, X 160],(6) Higher magnification to show the thickened epithelial layer (white asterisk) and excess hair follicles (black arrows) of active epithelium. H & E, X 400.

Rat dorsal skin of Gp3 (1) Upper Coagulative necrotic epidermis (black arrow) and numerous hair follicles with attached hyperplastic sebaceous glands (white arrow).[H & E, X 250], (2) Lower deep dermis

contained hyperplastic sebaceous glands (red arrow) at the bulge end of dilated hair follicles (black arrows) within a rich fatty connective tissue[H&E, X 250], (3) Thickened prickle (asterisk) and keratinocytic cell (black arrow) layers of the epidermis with numerous dermal papillae into the epidermis.[H & E, X 400], (4) Higher magnification to show the keratinized epidermal folding (white arrows) and dermal tissue (asterisk) with excess mononuclear cells[H & E, X 400.], (5) Area of mononuclear cell infiltrations and aggregations in the dermal fibrous connective tissue (red arrow)[H & E, X 400], (6) Cross section in increased variable sized upper hair follicles (white arrow) and other deep hair follicles (black arrows) that appeared surrounded by excess adipocytes [H & E, X 250].

Rat dorsal skin of Gp4 (1) Mostly thick epidermal epithelium (black asterisk), dermal fibrous connective tissue (white asterisk) with hair follicles (black arrow)[H & E, X 160], (2) Higher magnification to show some dermal contents of small duct of hair follicles (black arrow) as well as mononuclear cells (red arrow)[H & E, X 400],(3) The dermis formed mainly of fibrous connective tissue (asterisk) rich in excess congested blood capillaries (arrow)[H & E, X 160], (4)Higher magnification to show the dermal fibrous connective tissue with congested small capillaries (arrow) and upper thin epidermal epithelium (asterisk)[H & E, X 400], (5) Higher magnification to show the deep dermal fibrous connective tissue with excess of congested vasculatures (arrows)[H & E, X 400.],(6) Higher magnification to show the deep fatty dermal tissue (asterisk) with excess of pyknotichair follicles (arrows)[H & E, X 400].

Rat dorsal skin of Gp5 (1) Area of thick Coagulative necrotic epidermal and dermal tissue with excess numbers of inflammatory cell (asterisk), underlying damaged hair follicles (black arrows) and hemorrhages (white arrow)[H & E, X 250], (2) Area of loosely or liquefied necrotic dermal tissue with excess numbers of inflammatory cell and hemorrhages (asterisk), damaged hair follicle (white arrow) and focused on mainly bacterial clusters (black arrow) [H & E, X 250], (3) Higher magnification to show the

smaller slightly damaged hair follicles (black arrows) under a hyper-keratinized (white arrow) of the epidermis[H & E, X 400], (4) Higher magnification to show few numbers of sebocytes around a small slightly damaged hair follicle (black arrow) in addition to a group of microorganismal cluster (white arrow)[H & E, X 400], (5)The deeply present, slightly normal hair follicles surrounded by some fat cells (black arrows), hemorrhages and inflammatory cell infiltration in the dermal and hypodermal tissues (white arrows)[H & E, X 250], (6) Higher magnification to show the dermal and hypodermal adipose tissue (red arrow) and congested vasculatures (black arrows)[H & E, X 400].

Rat dorsal skin of Gp6 (1)Nearly thickened surface epidermal epithelium with numerous keratinized folds (white arrow) with appearance of excess numbers of variable sized hair follicles some appeared with dilated duct and less or no sebaceous glands (black arrows)[H & E, X 160], (2)Higher magnification to show the thickened epithelium (white asterisk) and keratinized infolding (white arrow) of the epidermis in addition to small hair follicles (black arrows) and numerous mononuclear cells in the dermal tissue (black asterisk)[H & E, X 400.], (3) Higher magnification to show the excess variable sized hyperplastic hair follicles with active epithelialization (black arrows) and mononuclear cell infiltration in the dermal tissue (asterisk)[H & E, X 400](4) Higher magnification to show the excess and keratin and hair (black asterisk) filling the infolding of the upper epidermal epithelium (arrow) [H & E, X 400], (5) Higher magnification to show mononuclear inflammatory cell infiltration in between the deep dermal adipocytes (arrow) in addition to the compacted hair follicles (astrisks)[H&E, X 400], (6)Congestion blood capillaries (white arrows) in the hypo-dermal fibrous connective tissue (black arrow). H & E, X 400.

Rat dorsal skin of Gp7 (1) Dermal tissue contained excess of hyperplastic hair follicles (white arrows) in addition to other hyperplastic active secretory sebaceous glands (black arrows)[H & E, X 160], (2) Higher magnification to show the hyperplastic active cells of the sebaceous glands (asterisk)

accompanied with small ductules of hair follicle (arrows) at the border[H&E, X 400], (3) Thick epidermal epithelium with keratinized infoldings (white arrows), sub-epithelial hair follicle (black arrow) in the fibrous connective tissue of the dermis (asterisk)[H&E, X 400], (4) Numerous thick walled empty ductules of hair follicles (white arrow) in the fibrous connective tissue of the dermis (asterisk)[H & E, X 400],(5) Dermal contents of thick walled empty ductules of hair follicles (arrows) in addition to enlarged active secretory sebaceous glands (red asterisks)[H & E, X 400], (6) Dermal fibrous connective tissue elements with congested blood capillaries (white arrows) and one damaged hair follicle[H & E, X 400].

Rat dorsal skin of Gp8 (1) Enlarged epidermal in fold contain excess compact keratin (black arrow) and congested vasculatures (red arrows) in the dermis (asterisk)[H & E, X 160], (2) Higher magnification to show the hyper-keratinization in the epidermal infold (arrow) and under fibrous connective tissue of the dermis (asterisk)[H & E, X 400], (3) Normal and somewhat thick epidermis (red arrow) accompanied with excess variable sized dermal contents of compacted hair follicles (black arrows)[H & E, X 250], (4) Higher magnification to show the nearly thick epidermis (red arrow) and small sized ductules of hair follicle (black arrow)[H & E, X 400], (5) Area of thick epidermal epithelium (asterisk) and dermal thick wall follicles (arrow) of hair follicles[H & E, X 400], (6)Excess variable sized hair follicles (arrows) in the deep dermal loose connective tissue[H & E, X 400].

Table S1: comparable values of contact angel and tensile strength of the MNS with other PVA based scaffold

**Scaffold**	**Contact angle /°**	**Tensile strength (MPa)**	**References**
Electrospun MNS	40.36	3	This work
Electrospun PVA/GO	35-55	2-5	[15]
Electrospun PVA/Epidermal	66	9-26	[33]
Electrospun PVA/Chitosan/Kaolin	63.54	2.93	[34]
Electrospun PVA/zein/SbQ	100-130	5-14	[35]

Table S2: Effect of different treatments on various biochemical parameters

Biochemical Parameters	Group 1	Group 2	Group 3	Group 4	Group 5	Group 6	Group 7	Group 8
**Total protein (g/L)**	**69.1±3.84^{c}**	**66.0±3.76^{d}**	**80.0±3.49^{b}**	**82.2±4.53^{a}**	**70.3±3.65^{b}**	**70.3±0.81^{c}**	**61.2±7.68^{d}**	**65.2±4.16^{d}**
**Albumin (g/L)**	**36.0±0.05^{a}**	**29.2±1.16^{b}**	**25.4±0.36^{c}**	**24.3±1.87^{d}**	**29.1±0.06^{b}**	**27.1±0.63^{b}**	**28.2±0.23^{b}**	**32.3±0.29^{b}**
**Creatinine (mg/dl)**	**0.85±0.03 ^{a}**	**0.95±0.03 ^{a}**	**0.96±0.04 ^{a}**	**0.94±0.03 ^{a}**	**0.90±0.04 ^{a}**	**0.96±0.02 ^{a}**	**0.90±0.02 ^{a}**	**0.92±0.04 ^{a}**
**GGT(U/L)**	**18.9±0.67 ^{a}**	**20.7±0.40 ^{a}**	**21.1±0.83 ^{a}**	**20.3±1.92 ^{a}**	**17.8±1.58 ^{a}**	**19.7±1.00 ^{a}**	**20.5±1.41 ^{a}**	**21.1±1.19 ^{a}**
**ALP(U/L)**	**58.9±1.88 ^{a}**	**62.2±$^{0.85a}$**	**64.5±1.22 ^{a}**	**62.0±1.68 ^{a}**	**59.1±0.96 ^{a}**	**65.3±1.07 ^{a}**	**67.2±1.26 ^{a}**	**59.1±1.43 ^{a}**
**ACP(U/L)**	**6.96±0.14 ^{a}**	**7.27±0.07 ^{a}**	**7.03±0.13 ^{a}**	**7.18±0.10 ^{a}**	**7.58±0.16 ^{a}**	**7.80±0.19 ^{a}**	**7.60±0.21 ^{a}**	**7.62±0.22 ^{a}**
**ALT(U/L)**	**30.3±1.56 ^{a}**	**34.5±1.53 ^{a}**	**32.0±1.27 ^{a}**	**29.4±0.89 ^{a}**	**29.5±0.41 ^{a}**	**34.3±0.85 ^{a}**	**30.8±0.99 ^{a}**	**33.7±1.47 ^{a}**
**AST(U/L)**	**26.9±1.31 ^{a}**	**27.3±0.94 ^{a}**	**27.9±9.68 ^{a}**	**28,1±1.83 ^{a}**	**30.7±0.98 ^{a}**	**29.2±1.84 ^{a}**	**29.6±1.31 ^{a}**	**30.8±1.80 ^{a}**
**LDH(U/L)**	**161±1.04^{d}**	**158±1.35^{d}**	**156±1.94^{d}**	**141±1.91^{e}**	**201±3.11^{a}**	**189±2.48^{b}**	**171±2.00^{c}**	**172±2.82^{c}**

*Data are presented as Mean ± S.E, S.E: Standard Error.
**Mean values within a row not sharing a common superscript letters (a, b, c, d, e) were significantly different, $p<0.05$.

Table S3: Effect of different treatments on hematological parameters

Parameters	Group1	Group2	Group3	Group4	Group5	Group6	Group7	Group8
**WBCs (10^3/ul)**	**19.4±0.36^c**	**29.4±0.39^a**	**17.3±0.23^d**	**26.0±2.70^b**	**28.4±1.12^b**	**29.3±0.89^a**	**23.2±2.19^c**	**23.0±1.66^c**
**Lymp#(10^3/ul)**	**15.5±0.24^c**	**23.2±0.10^b**	**12.6±0.30^d**	**20.4±2.35^c**	**24.0±0.95^a**	**24.1±1.32^a**	**16.5±1.01^c**	**18.0±1.10^c**
**Mon# (10^3/ul)**	**0.50±0.03^b**	**0.82±0.02^b**	**0.60±0.03^b**	**0.74±0.08^b**	**0.72±0.04^b**	**0.72±0.10^b**	**1.60±0.61^a**	**0.60±0.06^b**
**Gran# (10^3/ul)**	**3.42±0.43 ^{a}**	**5.44±0.35 ^{a}**	**4.00±0.22 ^{a}**	**4.54±0.52 ^{a}**	**4.94±0.19 ^{a}**	**4.52±0.60 ^{a}**	**5.00±0.76 ^{a}**	**4.34±0.57 ^{a}**
**Lymph (%)**	**80.1±2.12 ^{a}**	**78.4±0.95 ^{a}**	**73.4±1.30 ^{a}**	**79.0±2.19 ^{a}**	**80.1±0.52 ^{a}**	**81.5±2.76 ^{a}**	**73.2±3.07 ^{a}**	**78.6±1.54^a**
**Mon %**	**2.54±0.18^b**	**3.00±0.00^b**	**3.42±0.21^b**	**3.02±0.22^b**	**2.60±0.09^b**	**2.54±0.35^b**	**6.02±1.78^a**	**2.62±0.12^b**
**Gran%**	**17.1±1.97 ^{a}**	**18.2±0.94 ^{a}**	**22.5±1.16 ^{a}**	**17.6±1.98 ^{a}**	**17.2±0.45 ^{a}**	**15.4±2.41 ^{a}**	**20.4±1.31 ^{a}**	**18.3±1.42 ^{a}**
**RBCs (10^6/ul)**	**8.10±0.53 ^{a}**	**8.08±0.42 ^{a}**	**7.21±0.18 ^{a}**	**9.09±0.51 ^{a}**	**7.26±0.30 ^{a}**	**7.35±0.30 ^{a}**	**7.60±0.52 ^{a}**	**7.19±0.37 ^{a}**
**HGB (g/dL)**	**14.5±0.95 ^{a}**	**15.5±0.49 ^{a}**	**12.6±0.44 ^{a}**	**17.5±1.42 ^{a}**	**14.1±0.54 ^{a}**	**14.5±0.68 ^{a}**	**15.2±0.38 ^{a}**	**14.3±0.36 ^{a}**
**HCT %**	**44.6±2.61^a**	**49.1±1.33^a**	**38.5±1.08^b**	**52.3±4.62^a**	**45.0±2.01^a**	**44.3±1.59^a**	**52.1±2.05^a**	**45.5±1.08^a**
**MCV (fL)**	**55.2±1.12^a**	**61.4±3.56 ^{a}**	**52.5±0.38 ^{a}**	**56.5±1.85 ^{a}**	**60.3±0.50 ^{a}**	**58.4±0.20 ^{a}**	**67.5±7.01 ^{a}**	**63.2±2.58 ^{a}**
**MCH (pg)**	**18.2±0.33 ^{a}**	**19.5±1.07 ^{a}**	**17.3±0.19 ^{a}**	**19.2±0.44 ^{a}**	**19.1±0.14 ^{a}**	**19.3±0.22 ^{a}**	**19.3±0.79 ^{a}**	**20.0±0.86 ^{a}**
**MCHC (g/dL)**	**33.0±0.13 ^{a}**	**32.1±0.16 ^{a}**	**33.2±0.08 ^{a}**	**34.1±0.50 ^{a}**	**31.4±0.34 ^{a}**	**33.2±0.27 ^{a}**	**29.5±1.66 ^{a}**	**31.4±0.17 ^{a}**
**RDW %**	**16.3±0.61 ^{a}**	**16.1±0.32 ^{a}**	**15.5±0.50 ^{a}**	**15.5±0.38 ^{a}**	**19.4±0.35 ^{a}**	**17.3±0.47 ^{a}**	**23.6±4.87 ^{a}**	**17.0±0.13 ^{a}**

*Data are presented as Mean ± S.E, S.E: Standard Error.

**Mean values within a row not sharing a common superscript letters (a, b, c, d) were significantly different, p<0.05.

Table S4: Primer sequences used for real-time PCR analysis

**Gene**	**Primer**	**Sequence**
IL1R1	F	GTTTTTGGAACACCCTTCAGCC
	R	ACGAAGCAGATGAACGGATAGC
IL6	F	TCCTACCCCAACTTCCAATGCTC
	R	TTGGATGGTCTTGGTCCTTAGCC
TNF	F	AAATGGGCTCCCTCTCATCAGTTC
	R	TCTGCTTGGTGGTTTGCTACGAC
COX	F	TGTATGCTACCATCTGGCTTCGG
	R	GTTTGGAACAGTCGCTCGTCATC
